# Coumarin-Based Fluorescent Probes for Dual Recognition of Copper(II) and Iron(III) Ions and Their Application in Bio-Imaging

**DOI:** 10.3390/s140101358

**Published:** 2014-01-13

**Authors:** Olimpo García-Beltrán, Bruce K. Cassels, Claudio Pérez, Natalia Mena, Marco T. Núñez, Natalia P. Martínez, Paulina Pavez, Margarita E. Aliaga

**Affiliations:** 1 Department of Chemistry, Faculty of Sciences, University of Chile, Santiago 7800024, Chile; E-Mails: bcassels@u.uchile.cl (B.K.C.); claudio.perez.mendez@gmail.com (C.P.); 2 Facultad de Ciencias Naturales y Matemáticas, Universidad de Ibagué, Carrera 22 Calle 67, Ibagué 730001, Colombia; 3 Department of Biology, Faculty of Sciences, University of Chile, Santiago 7800024, Chile; E-Mails: npaz81@hotmail.com (N.M.); mnunez@uchile.cl (M.T.N.); 4 Facultad de Química, Pontificia Universidad Católica de Chile, Casilla 306, Santiago 6094411, Chile; E-Mails: natalia.dpma@gmail.com (N.P.M.); ppavezg@uc.cl (P.P.)

**Keywords:** Cu^2+^ ion, Fe^3+^ ion, turn-off probes, coumarin-based probes, fluorescence sensors, bio-imaging

## Abstract

Two new coumarin-based “turn-off” fluorescent probes, (*E*)-3-((3,4-dihydroxybenzylidene)amino)-7-hydroxy-2*H*-chromen-2-one (**BS1**) and (*E*)-3-((2,4-dihydroxybenzylidene)amino)-7-hydroxy-2*H*-chromen-2-one (**BS2**), were synthesized and their detection of copper(II) and iron(III) ions was studied. Results show that both compounds are highly selective for Cu^2+^ and Fe^3+^ ions over other metal ions. However, **BS2** is detected directly, while detection of **BS1** involves a hydrolysis reaction to regenerate 3-amino-7-hydroxycoumarin (**3**) and 3,4-dihydroxybenzaldehyde, of which **3** is able to react with copper(II) or iron(III) ions. The interaction between the tested compounds and copper or iron ions is associated with a large fluorescence decrease, showing detection limits of ca. 10^−5^ M. Preliminary studies employing epifluorescence microscopy demonstrate that Cu^2+^ and Fe^3+^ ions can be imaged in human neuroblastoma SH-SY5Y cells treated with the tested probes.

## 1. Introduction

Fluorescent probes may be defined as synthetic small molecules that react specifically with analytes to induce a marked change in their fluorescence properties; on the basis of such changes, the analytes can be determined [1–5]. These probes have been extensively investigated and widely used in many fields because of their powerful ability to improve analytical sensitivity, and in particular to be used in *in vivo* imaging studies. Of particular interest is the development of fluorescent probes for transition metal ions, such as Cu^2+^ and Fe^3+^, due to their biological relevance [6–8]. However, due to the low concentrations at which these metal ions are present in biosystems [9], high-sensitivity probes are necessary for practical applications. In recent years the literature has reported a large number of probes for Cu^2+^ and Fe^3+^ detection [10–13]. For the former ion, most of the probes involve a turn-off process, since copper ion often acts as a quencher via energy- or electron-transfer processes. However there are some probes designed on the basis of rhodamines, which show a fluorescence off-on response with reversible behavior upon complexation [14,15].

It is well known that Cu^2+^ can induce the hydrolysis of activated esters, Schiff bases, and hydrazones, which provides alternative approaches for the design of Cu^2+^ probes. These probes show a change in their fluorescence response to Cu^2+^ via Cu^2+^-promoted hydrolysis of the ester, imine or hydrazone function [16–18]. Interestingly, regarding Fe^3+^ ion detection, Lee *et al.* [19] have demonstrated that the strategy of a combination of Fe^3+^-induced Schiff-base hydrolysis and rhodamine spirolactam ring-opening in one system is an efficient model to achieve specific detection of Fe^3+^. Other fluorochromes with excellent photophysical properties, such as coumarin-based sensors, have also been reported for these metal ions [20]. However, in most of the cases studied, a high percentage of organic solvents is required due to the low water solubility of these probes. Thus, based on the fluorescent properties of coumarin derivatives and the importance of the presence of a Schiff base for the sensing mechanism of Cu^2+^ and Fe^3+^ ions, we have now synthesized, characterized and assessed two coumarin-based fluorescent probes for these biologically relevant ions, namely (*E*)-3-((3,4-dihydroxybenzylidene)amino)-7-hydroxy-2*H*-chromen-2-one (**BS1**) and (*E*)-3-((2,4-dihydroxybenzylidene)amino)-7-hydroxy-2*H*-chromen-2-one (**BS2**).

## 2. Experimental Section

### 2.1. Instruments and Reagents

All analytes were purchased from Sigma-Aldrich (Santiago, Chile) and were used as received. Unless indicated otherwise, all solutions employed in this study were prepared in Chelex-100-treated HEPES buffer (30 mM; pH 7.4). Melting points were determined on a Reichert-Jung Galen III hot-plate microscope equipped with a thermocouple. ^1^H-NMR spectra were recorded with a Bruker Avance 400 MHz spectrometer. All measurements were carried out in DMSO-*d*_6_. Absorption spectra were recorded at 25 °C using a Hewlett-Packard model HP 8453 instrument. The emission spectra were recorded at 25 °C on an Agilent Technologies Cary Eclipse fluorescence spectrophotometer. The fluorescence imaging was evaluated using a Zeiss Hal 100 epifluorescence inverted microscope.

### 2.2. Synthesis of the Probes

#### 2.2.1. (*E*)-3-((3,4-Dihydroxybenzylidene)amino)-7-hydroxy-2*H*-chromen-2-one (**BS1**)

3-Amino-7-hydroxy-2*H*-chromen-2-one (**3**, 0.56 g, 31 mmol) and 3,4-dihydroxybenzaldehyde (0.44 g, 31 mmol) were dissolved in absolute EtOH (10 mL) and refluxed for 2 h, during which a precipitate formed. After cooling, the product was collected and washed with an excess of hot EtOH to afford the product as a red solid, 0.93 g, 92%. m.p. > 320 °C. ^1^H-NMR (DMSO-*d*_6_): *δ* 10.46 (br, 1H, O-H), 10.17 (br, 1H, O-H), 9.87 (br, 1H, O-H), 8.80 (s, 1H, -N=CH-Ar), 7.72 (s, 1H, =C-H), 7.52 (d, 1H, Ar-H, *J* = 8.0 Hz) 7.49 (s, 1H, Ar-H), 7.30 (d, 1H, Ar-H, *J* = 8.0 Hz), 6.89 (d, 1H, Ar-H, *J* = 8.0 Hz), 6.80 (d, 1H, Ar-H, *J* = 8.0 Hz), 6.74 (s, 1H, OCH_3_).

#### 2.2.2. (*E*)-3-((2,4-Dihydroxybenzylidene)amino)-7-hydroxy-2*H*-chromen-2-one (**BS2**)

3-Amino-7-hydroxy-2*H*-chromen-2-one (**3**, 0.56 g, 31 mmol) and 2,4-dihydroxybenzaldehyde (0.43 g, 31 mmol) were dissolved in absolute EtOH (10 mL), and treated as above to give a red solid, 0.91 g, 98%. m.p. > 320 °C. ^1^H-NMR, δ 13.36 (s, 1H, O-H- -O=C), 10.57 (br, 1H, O-H), 10.37 (br, 1H, O-H), 9.02 (s, 1H, -N=CH-Ar), 7.93 (s, 1H, =C-H), 7.52 (d, 1H, *J* = 8.6 Hz), 7.39 (d, 1H, *J* = 8.6 Hz), 6.81 (dd, 1H, *J* = 8.0, 2.0 Hz), 6.75 (s, 1H), 6.40 (dd, 1H, *J* = 8.0, 2.0 Hz), 6.28 (d, 1H, *J* = 2.0 Hz).

### 2.3. Association Constant (Benesi-Hildebrand Plot)

Fluorescence intensity data for the complexes were plotted according to the Benesi-Hildebrand equation [21]:

(1) $$1/(F-F_{0})=1/\{K_{\text{a}}\times (F_{\max}-F_{0})\times [{\text{M}}^{\text{n}+}]\}+1/(F_{\max}-F_{0})$$

where *K*_a_ is the stability constant for complex formation, *F_0_* is the fluorescence intensity of the probe at the emission λ_max_ in the absence of metal ion, for **BS1** at 458 nm (with 340 nm excitation), for **BS2** at 437 nm (with 364 nm excitation) and for **3** at 454 nm (with 336 nm excitation). *F* is the observed fluorescence intensity as a function of the metal concentration ([M^n+^]: Cu^2+^ or Fe^3+^ ions) and *F*_max_ is the maximal fluorescence intensity in the presence of an excess of these ions in solution (600 μM).

### 2.4. Calculation of the Fluorescence Quantum Yield

The fluorescence quantum yield was determined using quinine sulfate dissolved in 0.05 M H_2_SO_4_ ( $\Phi_{\text{f}}^{\text{r}}=0.49$) as standard and was calculated using Equation (2) [22,23]:

(2) $$\Phi_{\text{f}}^{\text{s}}=\frac{{\text{F}}^{\text{s}}{\text{f}}_{\text{r}}{\left({\text{n}}_{\text{s}}\right)}^{2}}{{\text{F}}^{\text{r}}{\text{f}}_{\text{s}}{\left({\text{n}}_{\text{r}}\right)}^{2}}\Phi_{\text{f}}^{\text{r}}$$

where $\Phi_{f}^{s}$ and $\Phi_{f}^{r}$ are photoluminescence quantum yields and the subscripts s and r denote sample and reference, respectively. F^s^ and F^r^ are the integrated intensities (area under the curve) of sample and reference spectra, respectively. The terms f_r_ and f_s_ represent the absorption factors for sample and reference, respectively, *i.e.*, f_x_ = 1−10^−Ax^ (where the term A is the absorbance). Finally, η is the refractive index of the medium.

### 2.5. Computational Details

Optimization calculations were performed to find the ground state, transition state, intermediate and reaction product structures for **BS1** and **BS2**. The systems were optimized using the M05–2X method and 6-311G(d,p) basis set. The same method was used with Cu(II), but the LANL2DZ basis set was included. All other atoms of the molecule (C, H, O and N) and structures were optimized using the GAUSSIAN 03 suite of programs [24].

### 2.6. Cell Culture and Fluorescence Imaging for Cu^2+^

Human neuroblastoma SH-SY5Y cells (CRL-2266, American Type Culture Collection, Rockville, MD, USA) were cultured in MEM-F12 medium supplemented with 10% FBS, non-essential amino acids, antibiotic-antimycotic mixture, and 20 mM HEPES buffer, pH 7.2. The medium was replaced every 2 days. Cells were washed and the basal fluorescence was measured. They were then treated with the tested compounds (5 μM, 20 min) and washed with FBS, after which their fluorescence was determined. The cells were then incubated with Cu-His (200 μM, 15 min). The fluorescence was measured using an epifluorescence microscope at 63× amplification [25].

### 2.7. Cell Culture and Fluorescence Imaging for Fe^3+^

SH-SY5Y cells were cultured as described above. The cells were exposed to 20 μM Fe-NTA for 24 h and then incubated with the tested compounds (10 μM, 20 min). The fluorescence was measured as before.

## 3. Results and Discussion

### 3.1. Synthesis of **BS1** and **BS2**

**Scheme 1.**
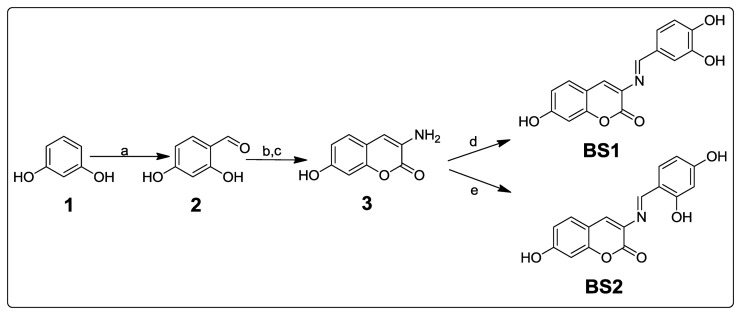
Synthetic route to **BS1** and **BS2**. *Reagents and conditions*: (**a**) POCl_3_, DMF, acetonitrile, 0–5 °C, 2 h; (**b**) acetylglycine, acetic anhydride, anhydrous sodium acetate, reflux 4 h; (**c**) 2:1 HCl/H_2_O reflux, 2 h; (**d**) 3,4-dihydroxybenzaldehyde; (**e**) 2,4-dihydroxybenzaldehyde, EtOH, reflux, 4 h.

As shown in Scheme 1, resorcinol (**1**) was formylated (Vilsmeier-Haack conditions) giving 2,4-dihydroxybenzaldehyde (**2**), which was subsequently condensed (Knoevenagel reaction) with acetylglycine and hydrolysed in one step to afford 3-amino-7-hydroxycoumarin (**3**). The coumarin was condensed with 3,4-dihydroxybenzaldehyde or 2,4-dihydroxybenzaldehyde to obtain **BS1** and **BS2**, by analogy with a literature procedure [26,27].

### 3.2. Spectral Characterization Studies

The compounds were characterized by ^1^H-NMR (in DMSO-*d*_6_), UV-Vis and fluorescence spectroscopy, the latter (in aqueous solution) as described in the Experimental section.

**Figure 1.**
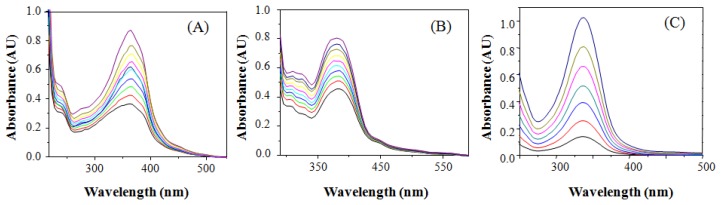
Absorption spectra of (**A**) **BS1**; (**B**) **BS2**; and (**C**) compound **3**; all in aqueous solution (30 mM HEPES buffer, pH 7.4, 1% DMSO).

Figure 1A,B shows the absorption spectra of **BS1** and **BS2**. The former displays a well-defined band at 360 nm (molar extinction coefficient of 22,830 M^−1^ cm^−1^). In the case of **BS2** its absorption spectrum exhibits a well-defined band at 364 nm (molar extinction coefficient of 18,600 M^−1^ cm^−1^).

The emission spectra were recorded by exciting **BS1** and **BS2** at 360 nm and 364 nm, respectively. To obtain the excitation spectra, the emissions were fixed at 458 nm and 437 nm, respectively, as shown in Figures S1 and S2 (Supplementary Data).

The Stokes shift values (the differences between excitation and emission maxima) were calculated from spectral data and are given in Table 1.

**Figure 2.**
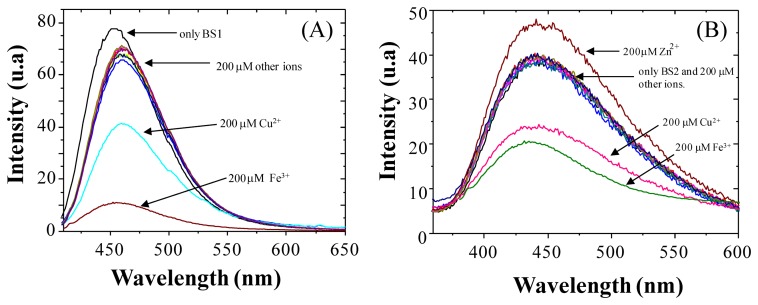
Change in fluorescence intensity of (**A**) **BS1** (2 μM) and (**B**) **BS2** (2 μM) upon addition of various metal ions (200 μM) (Fe^2+^, Fe^3+^, Ca^2+^, Co^2+^, Mg^2+^, Mn^2+^, Zn^2+^, Cd^2+^, Pb^2+^ and Hg^2+^).

To examine the molecular recognition of a variety of different metal cations by **BS1** and **BS2** we conducted fluorescence spectroscopy studies. As shown in Figure 2A,B, the fluorescence exhibited by each compound decreases in the presence of Cu^2+^/Fe^3+^ ions. It is important to note that other metal ions of interest (at 200 μM concentration) failed to show any significant interference at 458 nm for **BS1** and at 437 nm for **BS2**. However, for **BS2** a slight fluorescence increase was observed when a concentration of 200 μM of Zn^2+^ was added.

**Figure 3.**
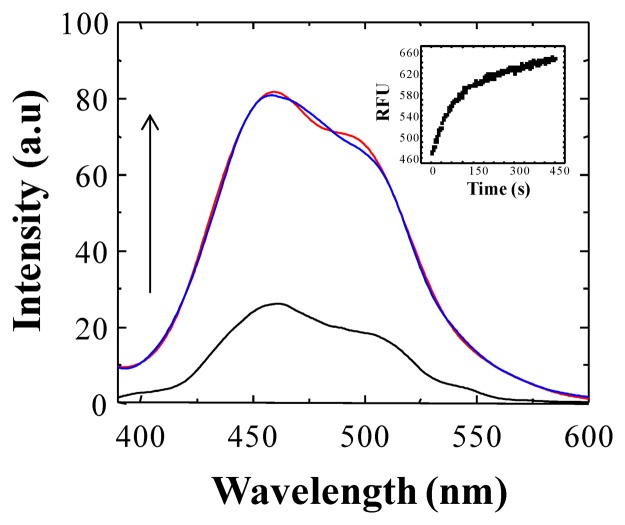
Fluorescence spectra of **BS1** recorded at different times. The black line represents the fluorescence intensity of a freshly prepared solution of **BS1** (2 μM) in 30 mM HEPES buffer, pH 7.4, 1% DMSO; the blue line represents the fluorescence intensity of the same solution after 360 s of incubation; and the red line represents the fluorescence intensity of the same solution after 720 s of incubation. Excitation at 340 nm (slit = 5.0/5.0). Inset: Time-dependent fluorescence spectra of **BS1** (2 μM) at 25 °C, λ_exc_ = 340 nm, *t =* 0–450 s.

Considering the ability of **BS1** and **BS2** to interact with free Cu^2+^ or Fe^3+^ ions in aqueous solution, we assessed the effect of the addition of increasing concentration of these ions on the fluorescence intensity of **BS1** and **BS2**. Controls conducted with solutions containing either **BS1** or **BS2** showed that, under the conditions of the assay (e.g., incubation at 25 °C during 360 s), **BS1** exhibits–in the absence of metal- an almost threefold increase of its fluorescence intensity (see Figure 3). In the case of **BS2** no changes in fluorescence intensity were observed under identical experimental conditions (not shown).

In view of the results presented in Figure 3 and considering the reported susceptibility to hydrolysis of compounds containing a Schiff base, we decided to evaluate the possibility that **BS1** might, in addition to its sensing action, be decomposing in the buffered aqueous medium. With the aim of elucidating the chemical nature of the compound(s) that might be arising during the incubation of a **BS1** solution, we conducted suitable ^1^H-NMR experiments.

**Figure 4.**
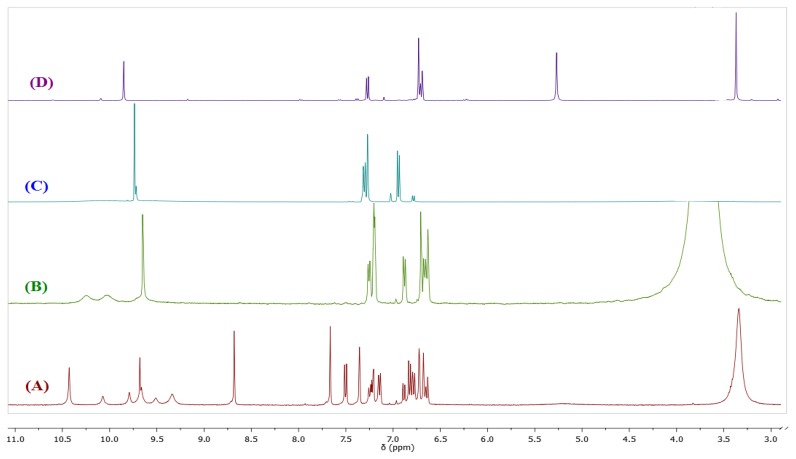
(**A**) ^1^H-NMR spectra (DMSO-*d*_6_) of **BS1**; (**B**) **BS1** after adding water; (**C**) 3,4-dihydroxybenzaldehyde and (**D**) 3-amino-7-hydroxy-2*H*-chromen-2-one (**3**).

Figure 4 depicts the spectra of **BS1** (1 mM in part A), when adding 10% of water to **BS1** (part B), and of its precursors, 3,4-dihydroxybenzaldehyde (part C) and 3-amino-7-hydroxy-2*H*-chromen-2-one (**3**) (part D). Spectrum (B) shows the disappearance of some characteristic resonances of **BS1** and depicts features that are present in both spectra (C) and (D). Based on these NMR results we propose that **BS1** indeed undergoes hydrolysis giving rise to its precursors, *i.e.*, 3-amino-7-hydroxy-2*H*-chromen-2-one (**3**) and 3,4-dihydroxybenzaldehyde.

**Scheme 2.**
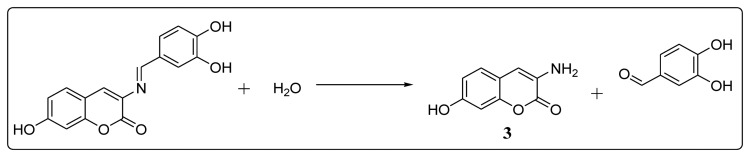
Decomposition reaction proposed for **BS1**.

In line with the previous observation, the fluorescence spectra of **BS1** (Figure S1) show an excitation band at 340 nm and an emission band at 461 nm, spectral features that are practically indistinguishable from those presented by the precursor **3** (Figure S3). The latter result is consistent with the NMR data and strongly suggests that the compound formed by decomposition of **BS1** in solution is 3-amino-7-hydroxy-2*H*-chromen-2-one (**3**), as indicated in Scheme 2.

Regarding the stability of precursor **3**, it is important to note that its NMR spectrum recorded after 10 h of incubation with added water is identical to that obtained for the freshly prepared solution in DMSO-*d*_6_ (see Supplementary Data, Figure S4).

The stability of **3** in aqueous solution suggests that this compound might be the substance actually involved in the Cu^2+^/Fe^3+^ ion detection presented in Figure 2A. Therefore, we focused our study further on evaluating whether the fluorescence intensity of **3** might decrease as a result of its interaction with these metal ions.

**Figure 5.**
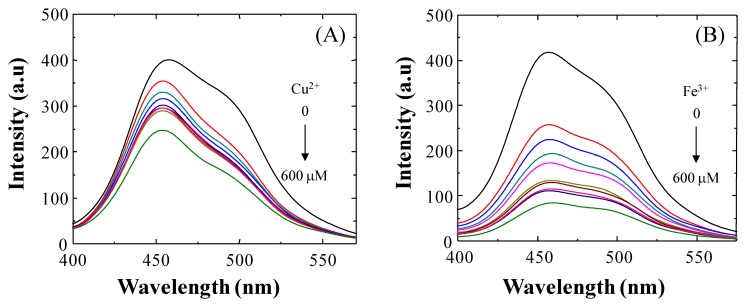
(**A**) Fluorescence spectra (2 μM) of **3** recorded upon the addition of copper ion (0–300 equiv.) in aqueous solution (30 mM HEPES buffer, pH 7.4, 1% DMSO). Excitation at 340 nm (slit = 5.0/5.0); (**B**) Fluorescence spectra (2 μM) of **3** recorded upon the addition of iron ion (0–300 equiv.) in aqueous solution (30 mM HEPES buffer, pH 7.4, 1% DMSO). Excitation at 340 nm (slit = 5.0/5.0).

As shown in Figure 5, upon incremental addition of Cu^2+^ ion (0–300 equiv.) to a solution containing **3**, the fluorescence emission is gradually quenched and reaches the saturation state when 300 equiv. of Cu^2+^ ion are employed (not shown). This fluorescence quenching of **3** may occur by excitation energy transfer from the ligand (probe) to the metal d-orbital and/or LMCT [28]. A similar quenching of fluorescence was observed when Fe^3+^ ion was tested. In fact, when 600 μM of Fe^3+^ ion was added to a solution of **3**, a quenching efficiency of (I_0_ − I)/I_0_ × 100 = 79.80% was observed at 454 nm. Based on the evidence presented here, we propose that under our experimental conditions **3** is a good probe for detecting both Cu^2+^ and Fe^3+^.

**Figure 6.**
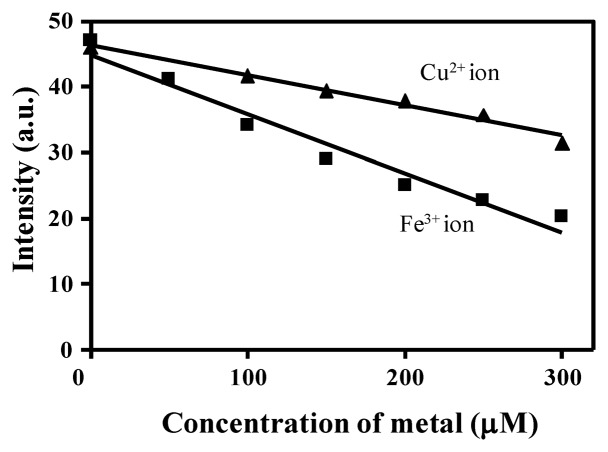
Fluorescence responses of **BS2** (2 μM) in the presence of copper (▲) or iron (■) ions (0–300 μM) in aqueous solution (30 mM HEPES buffer, pH 7.4, 1% DMSO). Excitation at 340 nm (slit = 5.0/5.0).

Recently, other authors [29] have reported the importance of the presence of an *o*-OH group in the benzylidene moiety of the Schiff base, which serves as an additional binding site for Cu^2+^ ion coordination to provide a stable complex. In view of the latter and the results presented above related to the hydrolysis of **BS1**, we propose that the *o*-OH unit of **BS2**, by forming an intramolecular hydrogen bond, makes **BS2** more resistant to this decomposition reaction. In fact, we observed that the NMR spectrum of **BS2** remains unaltered after its exposure to water or a long incubation time (Figure S5). Therefore, considering the stability of **BS2**, we also characterized the sensitivity of this probe toward Cu^2+^ and Fe^3+^ ions in aqueous medium. The results are presented in Figure 6. At pH 7.4 a decrease in the fluorescence emission intensity of **BS2**, dependent on the metal concentration, was observed at 437 nm upon addition of Fe^3+^ ions and a smaller decrease was seen after adding Cu^2+^ ions (Figure 6). Quenching efficiencies of (I_0_ − I)/I_0_ × 100 = 31.53% and 56.76% for Cu^2+^ and Fe^3+^ ion, respectively, were determined at 437 nm.

Benesi-Hildebrand plots from fluorescence titration data of **BS1**, **BS2** and **3** with Cu^2+^ or Fe^3+^ ions were non-linear, indicating changes in the stoichiometry of the metal-containing complexes (data not shown). As can be seen in Table 2, in most cases the detection limits were ca. 5 × 10^−5^ mol/L, based on 3 × σ/*k* (where σ is the standard deviation of the blank solution and *k* is the slope of the calibration plot obtained from spectra data in Figures 2,5,S6). These values are similar to values reported in the literature for other Cu^2+^/Fe^3+^ probes [28,30].

### 3.3. Computational Study

**Figure 7.**
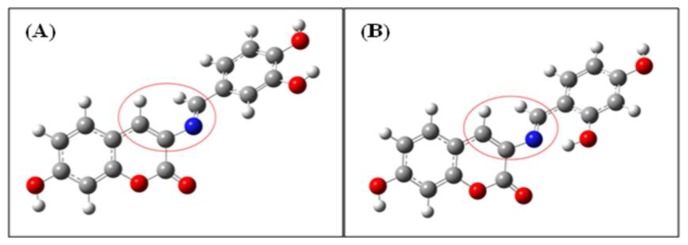
Calculated structures for (**A**) **BS1** and (**B**) **BS2**. Atoms forming dihedral angles are shown in red circles.

To assess the stability of **BS2** in comparison with **BS1**, a theoretical study within the framework of Natural Bond Orbital (NBO) analysis was carried out [31]. This procedure shows that the proposed intramolecular hydrogen bond for **BS2** has a distance of 1.738 Å and energy of 30.88 kcal mol^−1^. These values are within the established range for strong hydrogen bonds [32,33]. Therefore, the stability of **BS2** could be associated with this intramolecular interaction. In addition, Table 3 and Figure 7A,B shows that for **BS1** and **BS2** their dihedral angles (between atoms depicted inside the red circles in Figure 7) decrease from 44.22° for **BS1** to 41.03° for **BS2**. The latter could be another consequence of the presence of the intramolecular hydrogen bond formed between the hydrogen of the hydroxyl group and the nitrogen atom of the imine group present in **BS2**.

On the other hand, with the aim of understanding why **BS1** undergoes a decomposition reaction to regenerate its precursors, it is necessary to calculate the reaction profile. This profile is shown in Figure S7. This shows that the reaction proceeds through a stepwise mechanism, where the rate-determining step is the formation of the first transition state. The activation energies are shown in Table 3.

The data presented in Table 3 indicate that the first step for **BS2** requires slightly more energy than in the reaction of **BS1**, which suggest that the reaction should be faster for **BS1** than for **BS2**. Taking into consideration the latter and the stability of **BS2** (assessed under our experimental conditions), we pursued additional theoretical studies to investigate the binding of copper ion to **BS2**. As shown in Figure S8, the imine, carbonyl, and hydroxyl groups present in **BS2** can be important coordination sites for a copper ion.

### 3.4. Competitive Binding Studies

To examine the interferences of different metal ions with the recognition of Cu^2+^ or Fe^3+^ by **3** and **BS2**, fluorescence competition experiments were subsequently carried out. As shown in Figure S9A,B, the fluorescence intensity of **3** and **BS2** solutions, respectively, was not significantly quenched in the presence of the selected potential competitive metal ions, whereas subsequent addition of Fe^3+^ ions led to strong quenching. Similar results were obtained in the presence of Cu^2+^ ions (data not shown). These results demonstrate that the coexisting metal ion does not interfere significantly with Fe^3+^ or Cu^2+^ recognition.

### 3.5. Application of the Proposed Probes for the Detection of Copper or Iron Ions in Living Cells

**Figure 8.**
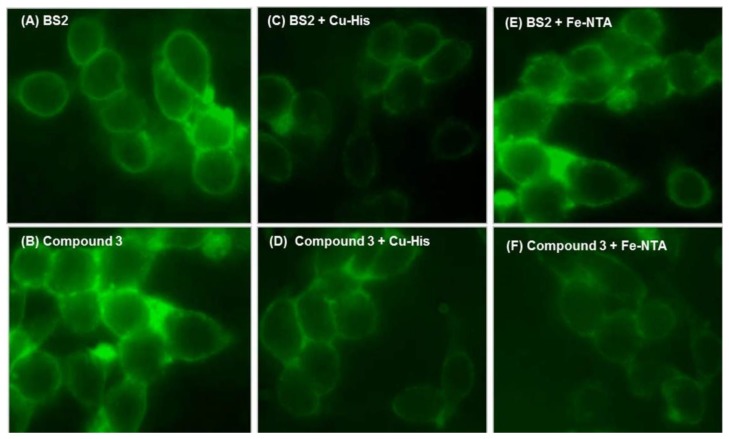
SH-SY5Y cells were washed and treated with compound **BS2** or **3** and the basal fluorescence was measured: (**A**) and (**B**), respectively. The cells were incubated with Cu-His (200 μM, 15 min) and their fluorescence determined (**C**) and (**D**). The cells were incubated with Fe-NTA (80 μM, 12 h) and their fluorescence determined (**E**) and (**F**). The fluorescence was measured using epi-fluorescence microscopy at 63× amplification.

To further demonstrate the practical applicability of the tested probes to detect Cu^2+^ and Fe^3+^ in living cells, the fluorescence images of SH-SY5Y cells were recorded before and after addition of Cu^2+^ and Fe^3+^ ions (Figure 8).

First, to determine the cell permeability of **BS2** or **3**, the cells were initially incubated either with **BS2** or **3**, under physiological conditions. Figure 8A,B shows that both probes have the ability to penetrate the cell and generate a fluorescent signal distributed throughout the cytoplasm. From recent work [34] showing the intracellular localization of fluorescent probes in living cells it is expected that **BS2** will be more uniformly distributed in the cytoplasm, while **3** would be expected to accumulate in lysosomes. After adding Cu^2+^-histidine complex as a source of Cu^2+^, a decrease in the fluorescence intensity is observed (Figure 8C,D). In the case of cells incubated with **BS2** the addition of Fe^3+^ as Fe-NTA complex was not associated with changes in the fluorescence intensity (Figure 8E). The latter result could be explained considering that **BS2** is unable to remove Fe^3+^ ion from the Fe-NTA complex, due to the high value of the Fe(III)-NTA stability constant (*K_a_* = 10^12^) [35].

On the other hand, when this assay was carried out using **3**, the fluorescent hydrolysis product of **BS1**, **3** accumulates within the cell (Figure 8B) and responds by fluorescence quenching to Cu^2+^ (Figure 8D) and Fe^3+^ (Figure 8F). This behavior is in accordance with the abovementioned results (Figure 5).

## 4. Conclusions/Outlook

Coumarin-based probes (compounds **BS1** and **BS2**) were synthesized and characterized for recognition of Cu^2+^/Fe^3+^. Our studies indicate that these compounds present high selectivity for Cu^2+^ and Fe^3+^ ions over other metal ions. However, the detection mode for such ions is different, being a direct reaction in the case of **BS2** and an indirect reaction with **BS1**. The latter involves a hydrolysis reaction to generate 3-amino-7-hydroxycoumarin (**3**) and 3,4-dihydroxybenzaldehyde, where **3** is the actual substance reacting with Cu^2+^ or Fe^3+^ ions and undergoing fluorescence quenching. On the basis of a theoretical study, a binding mode between **3** and Cu^2+^ is proposed. Finally, the applicability of the proposed probes was demonstrated in living cells with satisfactory results: **BS2** is suitable for the detection of Cu^2+^ ion while **3** allows dual recognition of Cu^2+^ and Fe^3+^ ions in biological systems.

## Supplementary Material



## Figures and Tables

**Table 1.** Emission and excitation spectrum-related data of tested compounds.

**Compound**	**UV-Vis**		**Fluorescence**			
	
	**λ_m__á__x_** **(nm)**	**ε** **(M^−1^ cm^−1^)**	**λ_exc_** **(nm)**	**λ_em_** **(nm)**	**Relative Quantum** **Yield** ( $\Phi_{\text{f}}^{\text{r}}$)	**Stokes'** **Shift (nm)**
**BS1**	360	22,830	340	458	nd	118
**BS2**	364	18,600	364	437	0.09	73
**3**	336	12,919	336	454	0.44	118

nd = not determined.

**Table 2.** Detection and quantification limit for each tested complex.

**Compound**	**Limit of Detection (mol/L)**	**Limit of Quantification (mol/L)**
BS1-Cu^2+^	1.27 × 10^−4^	4.22 × 10^−4^
BS1-Fe^3+^	5.17 × 10^−5^	1.72 × 10^−4^
BS2-Cu^2+^	1.04 × 10^−4^	3.45 × 10^−4^
BS2-Fe^3+^	4.87 × 10^−5^	1.62 × 10^−4^
3-Cu^2+^	5.41 × 10^−5^	1.80 × 10^−4^
3-Fe^3+^	5.03 × 10^−5^	1.68 × 10^−4^

**Table 3.** Dihedral angles and Gibbs free energies of activation for **BS1** and **BS2**.

**Reaction**	**Dihedral Angle**	ΔG*_1_ kcal/mol	ΔG*_2_ kcal/mol
**BS1**	44.22°	51.03	36.42
**BS2**	41.03°	52.84	37.15

## Nomenclature

**BS1**: (*E*)-3-((3,4-Dihydroxybenzylidene)amino)-7-hydroxy-2*H*-chromen-2-one
**BS2**: (*E*)-3-((2,4-Dihydroxybenzylidene)amino)-7-hydroxy-2*H*-chromen-2-one
FBS: Fetal bovine serum
MEM-F12: Modified Eagle Medium-F12
His: Histidine
LMCT: Ligand to metal charge transfer
HEPES: (4-(2-Hydroxyethyl)-1-piperazineethanesulfonic acid)
NTA: Nitrilotriacetic acid
DMSO: Dimethyl sulfoxide
DMF: Dimethyl formamide
Fe-NTA: Nitrilotriacetic acid-Fe(III)
