# Supplementary material for: Coumarin-Based Fluorescent Probes for Dual Recognition of Copper(II) and Iron(III) Ions and Their Application in Bio-Imaging
Source: Sensors (Basel). 2014 Jan 13;14(1):1358–71. doi: 10.3390/s140101358 (PMC3926618; doi:10.3390/s140101358)

OPEN ACCESS

sensors

ISSN 1424-8220

www.mdpi.com/journal/sensors

Supplementary Information

Coumarin-Based Fluorescent Probes for Dual Recognition of Copper(II) and Iron(III) Ions and Their Application in Bio-Imaging. *Sensors* 2014, *14*, 1385-1371

Olimpo García-Beltrán [1,2,*], Bruce K. Cassels [1], Claudio Pérez [1], Natalia Mena [3], Marco T. Núñez [3], Natalia P. Martínez [4], Paulina Pavez [4] and Margarita E. Aliaga [4,*]

1 Department of Chemistry, Faculty of Sciences, University of Chile, Santiago 7800024, Chile; E-Mails: bcassels@u.uchile.cl (B.K.C.); claudio.perez.mendez@gmail.com (C.P.)

2 Facultad de Ciencias Naturales y Matemáticas, Universidad de Ibagué, Carrera 22 Calle 67, Ibagué 730001, Colombia

3 Department of Biology, Faculty of Sciences, University of Chile, Santiago 7800024, Chile; E-Mails: npaz81@hotmail.com (N.M.); mnunez@uchile.cl (M.T.N.)

4 Facultad de Química, Pontificia Universidad Católica de Chile, Casilla 306, Santiago 6094411, Chile; E-Mails: natalia.dpma@gmail.com (N.P.M.); ppavezg@uc.cl (P.P.)

***** Authors to whom correspondence should be addressed; E-Mails: ojgarciab@ug.uchile.cl (O.G.-B.); mealiaga@uc.cl (M.E.A.); Tel.: +56-2-23547126 (M.E.A.); Fax: +56-2-23544744 (M.E.A.).

Supplementary Information

**Figure S1.** Fluorescence spectra of excitation (black color line) and emission (red color line) of BS1 (2 µM), λ_{Exc} = 340 nm, λ_{Em} = 458nm.	**S3**
**Figure S2.** Fluorescence spectra excitation (black color line) and emission (red color line) of **BS2** (2 µM), λ_{Exc} = 364 nm, λ_{Em} = 437 nm.	**S3**
**Figure S3.** Fluorescence spectra excitation (black color line) and emission (red color line) of 3 (2µM), λ_{Exc} = 336 nm, λ_{Em} = 454 nm.	**S3**
**Figure S4.** ^{1}H-NMR spectra of 3 in DMSO-*(d_6)* at 300 K. Red color represents t=0 and light blue color represents spectrum after water addition.	**S4**
**Figure S5.** ^{1}H-NMR spectra of BS2 at different incubation times. (A) Spectrum of a freshly prepared solution of BS2, (B) Spectrum of a solution containing BS2 after 120 min. of incubation and (C) Spectrum of a solution containing BS2 plus water.	**S4**
**Figure S6.** (A) Fluorescence spectra (2 µM) of BS1 recorded upon the addition of copper ion (0–300 equiv.) in aqueous solution (30 mM HEPES buffer, pH 7.4, 1% DMSO). Excitation at 340 nm (slit = 5.0/5.0). (B) Fluorescence spectra (2 µM) of BS1 recorded upon the addition of iron ion (0–300 equiv.) in aqueous solution (30 mM HEPES buffer, pH 7.4, 1% DMSO). Excitation at 340 nm.	**S5**
**Figure S7.** Reaction profile for the *auto-decomposition* reaction of BS1.	**S5**
**Figure S8.** Calculated structure for the binding modes for the complex formed between BS2 and copper ion.	**S5**
**Figure S9.** Changes in fluorescence intensity (expressed as Relative Fluorescence Units “RFU”) of solution 3 (2 µM) induced by different metal ions.	**S6**

Figure S1. Fluorescence spectra of excitation (black color line) and emission (red color line) of **BS1** (2 µM), λ_{Exc} = 340 nm, λ_{Em} = 458nm. Slit 5.0/5.0.

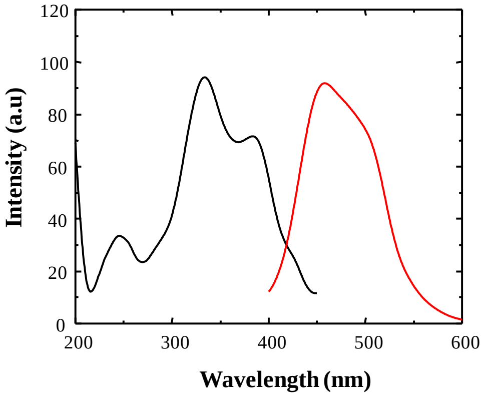

Figure S2. Fluorescence spectra excitation (black color line) and emission (red color line) of **BS2** (2 µM), λ_{Exc} = 364 nm, λ_{Em} = 437 nm. Slit 5.0/5.0.

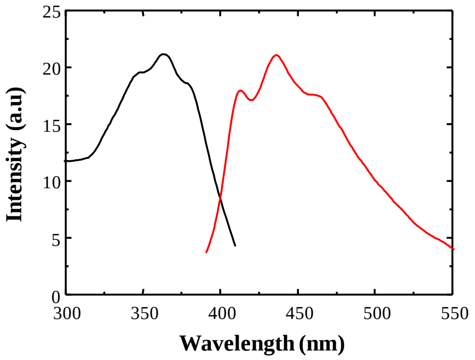

Figure S3. Fluorescence spectra excitation (black color line) and emission (red color line) of **3** (2 µM), λ_{Exc} = 336 nm, λ_{Em} = 454 nm. Slit 5.0/5.0.

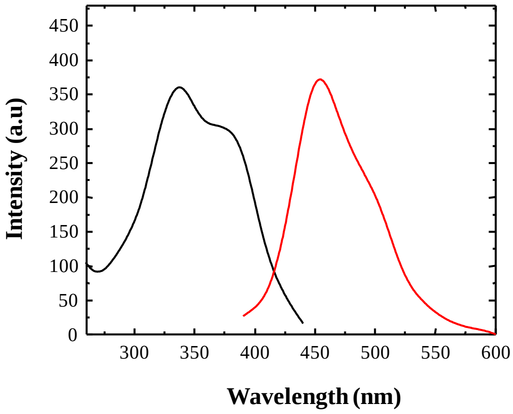

Figure S4. ^{1}H-NMR spectra of **3** in DMSO-(d_6) at 300 K. Red color represents t = 0 and light blue color represents spectrum after water addition (10 %).

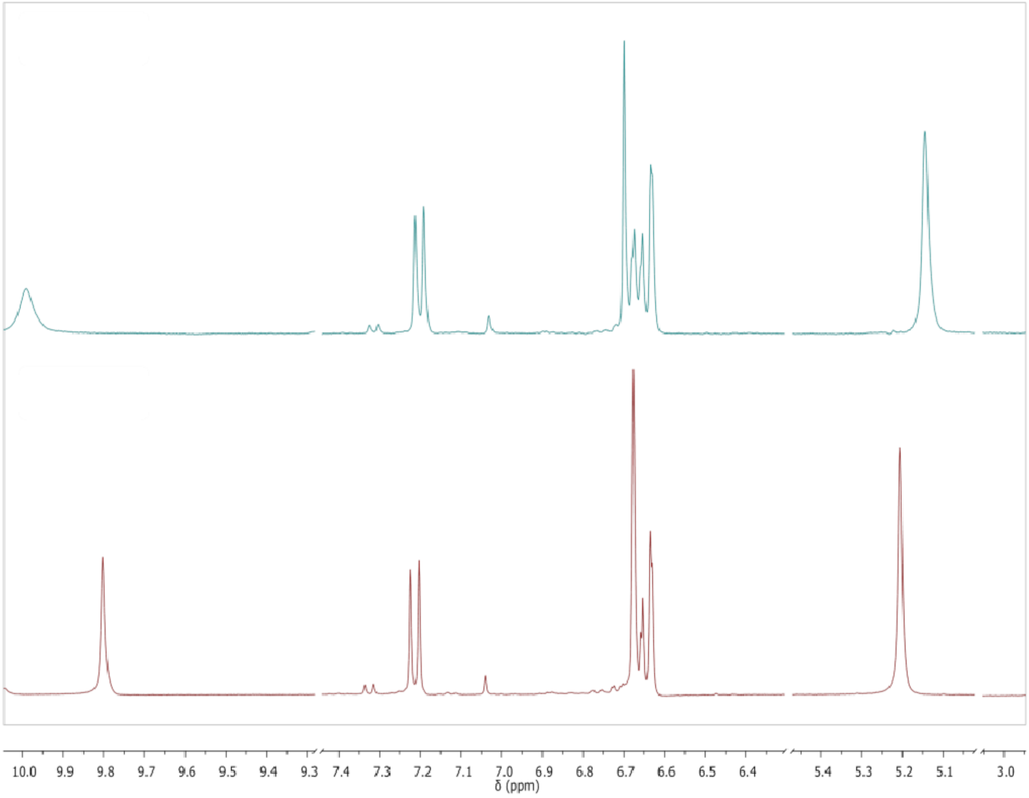

Figure S5. ^{1}H-NMR spectra of **BS2** at different incubation times. (**A**) Spectrum of a freshly prepared solution of **BS2**; (**B**) Spectrum of a solution containing **BS2** after 120 min. of incubation and (**C**) Spectrum of a solution containing **BS2** plus water.

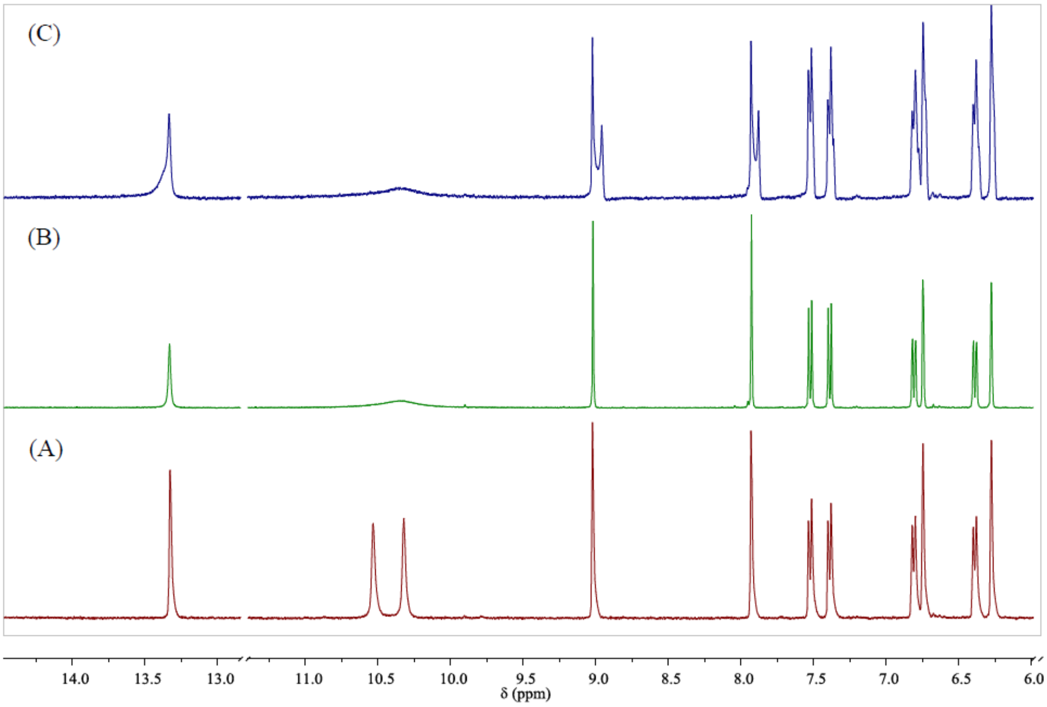

Figure S6. (**A**) Fluorescence spectra (2 μM) of **BS1** recorded upon the addition of copper ion (0–300 equiv.) in aqueous solution (30 mM HEPES buffer, pH 7.4, 1% DMSO). Excitation at 340 nm (slit = 5.0/5.0); (**B**) Fluorescence spectra (2 μM) of **BS1** recorded upon the addition of iron ion (0–300 equiv.) in aqueous solution (30 mM HEPES buffer, pH 7.4, 1% DMSO). Excitation at 340 nm (Slit = 5.0/5.0).

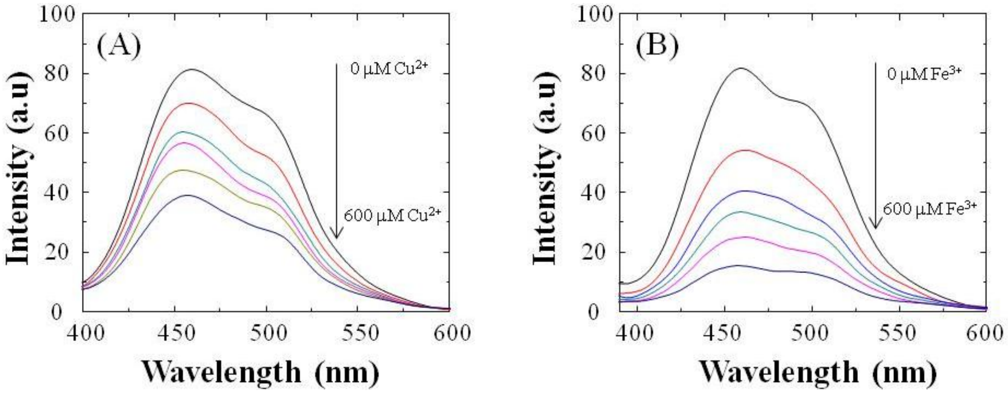

Figure S7. Reaction profile for the *auto-decomposition* reaction of **BS1**.

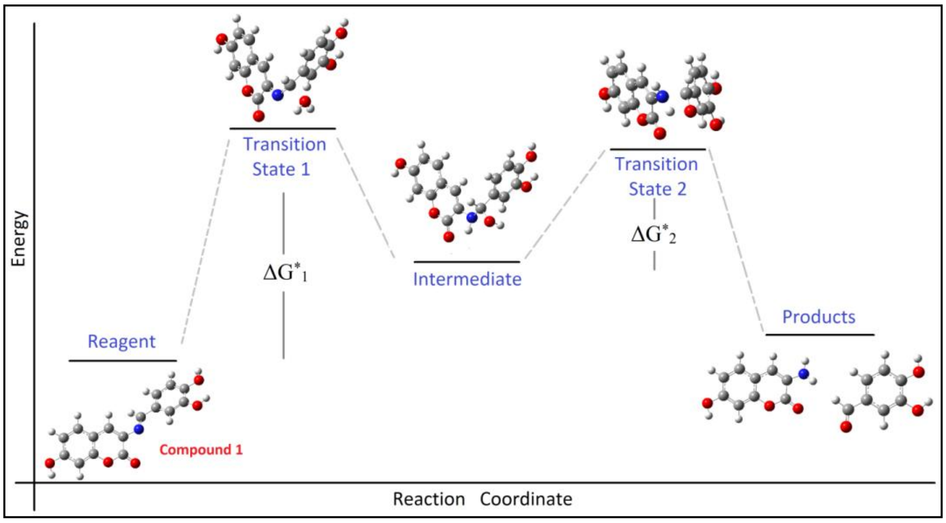

Figure S8. Calculated structure for the binding modes for the complex formed between **BS2** and copper ion.

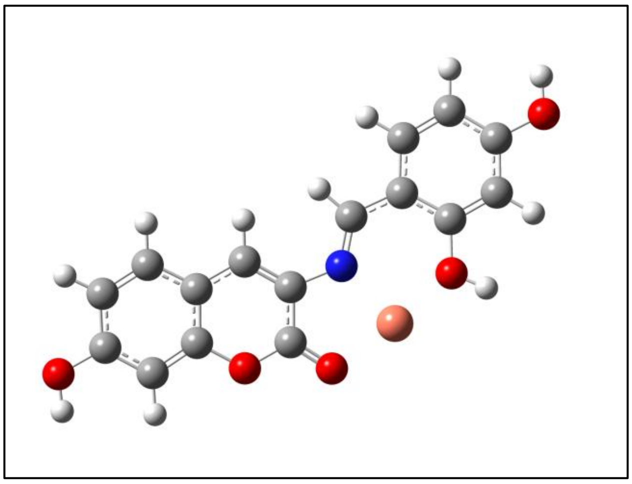

Figure S9. Changes in fluorescence intensity (expressed as Relative Fluorescence Units "RFU") of solution **3** (2 μM) induced by different metal ions. In graph (**A**) the black bars represent the fluorescence intensity due to **3** alone, grey bars represent the fluorescence intensity due to **3** plus 300 equiv. of miscellaneous metal ions and the white bars represent the fluorescence intensity of the above solution upon further addition of 10 equiv of Fe^{3+} (λ_{em} = 454 nm); In graph (**B**) the black bars represent the fluorescence intensity due to **BS2** alone, grey bars represent the fluorescence intensity due to **BS2** plus 300 equiv. of miscellaneous metal ions and the white bars represent the fluorescence intensity of the above solution upon further addition of 10 equiv of Fe^{3+} (λ_{em} = 437 nm).

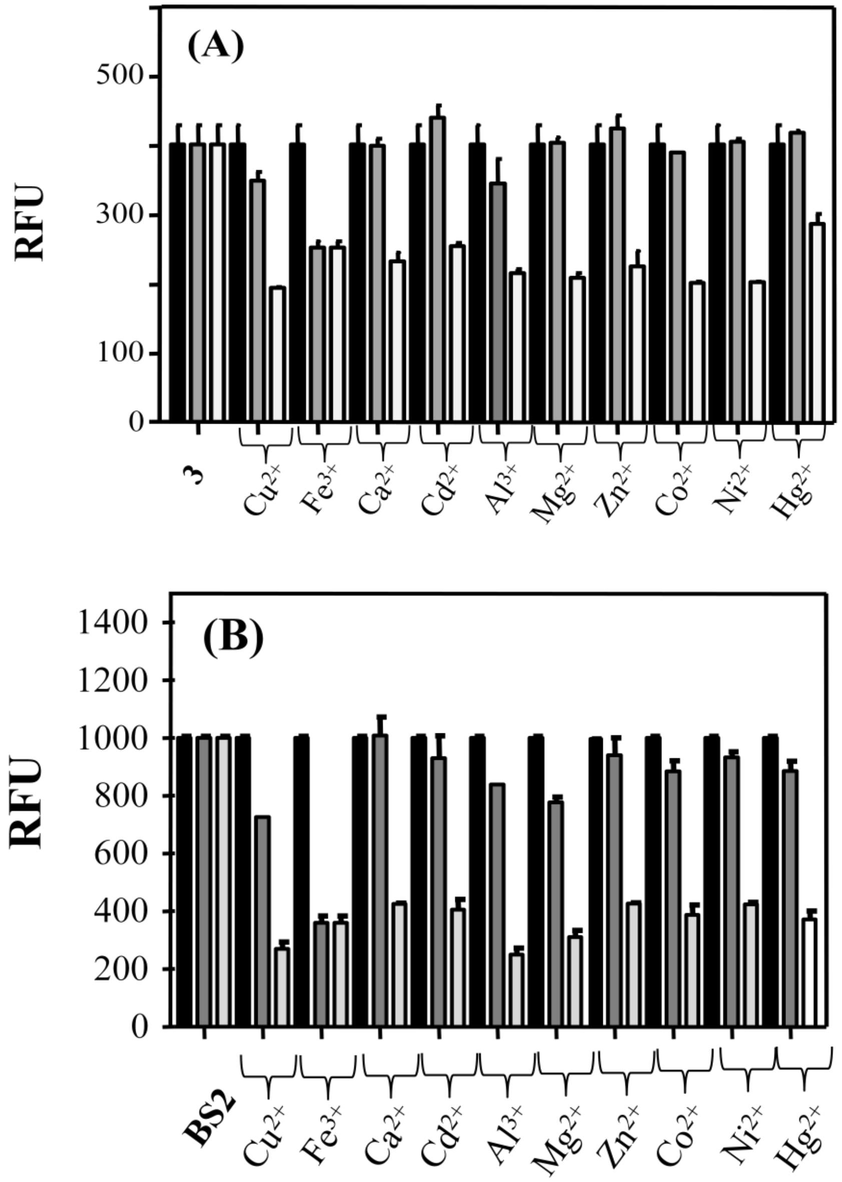

Supplement: Supplementary file 1 [file sensors-14-01358-s001.pdf]
